# PRAME immunohistochemistry compared to traditional FISH testing in spitzoid neoplasms and other difficult to diagnose melanocytic neoplasms

**DOI:** 10.3389/fmed.2023.1265827

**Published:** 2023-10-09

**Authors:** Elizabeth Warbasse, Darius Mehregan, Sarah Utz, R. Brent Stansfield, Judith Abrams

**Affiliations:** ^1^Department of Dermatology and Cutaneous Surgery, University of South Florida Morsani College of Medicine, Tampa, FL, United States; ^2^Department of Dermatology, Wayne State University School of Medicine, Detroit, MI, United States; ^3^Essentia Health, Duluth, MN, United States; ^4^Office of Graduate Medical Education, Wayne State University School of Medicine, Detroit, MI, United States; ^5^Wayne State University School of Medicine, Detroit, MI, United States

**Keywords:** PRAME, immunohistochemistry, dermatopathology, fluorescence *in situ* hybridization, Spitz, melanoma, melanocytic neoplasms

## Abstract

PRAME (PReferentially expressed Antigen in Melanoma) is a gene first identified in melanoma. It has been proposed as a useful marker to differentiate melanoma from benign melanocytic neoplasms. Recently genomic testing using fluorescence *in situ* hybridization has been used to aid in the diagnosis of difficult melanocytic neoplasms. We have compared PRAME staining to FISH testing results in 83 difficult to classify melanocytic neoplasms which showed spitzoid histologic features. A relatively low sensitivity of 29.6% and high specificity of 76.8% is seen with PRAME staining as compared to genomic testing with fluorescence *in situ* hybridization. This study highlights the limitations of PRAME staining in spitzoid neoplasms.

## 1. Introduction

PRAME (Preferentially expressed Antigen in Melanoma) is a gene that was first identified via analysis of genetic material from a melanoma patient in 1997; it is found in melanoma cells, as well as in the normal tissues of the testes, and to a smaller degree, endometrium, ovaries, and the adrenal glands (1). Squamous cell carcinomas of the lung, some sarcomas, and acute leukemias may also be positive for PRAME, which has led to its use as a potential target for immunotherapy (1, 2). In the field of dermatopathology, PRAME immunohistochemical stain has been proposed as a tool to identify melanoma cells in skin biopsies. This study aims to investigate the utility of PRAME immunohistochemical staining in difficult to diagnose melanocytic neoplasms, particularly spitzoid neoplasms.

Fluorescence *in-situ* hybridization (FISH) genomic testing in spitzoid and other difficult melanocytic neoplasms has been shown to have a sensitivity of up to 97.6% and a specificity of 72.7% (3). However, it is costly and time consuming, thus not always utilized in making the diagnosis of melanoma, which has long relied upon histopathology. FISH testing may be correlated with histopathologic assessment in these difficult to diagnose cases. Prior studies have demonstrated that the PRAME gene has been shown to be expressed in 13.6% of nonmalignant melanocytic nevi (4). This presents a potential pitfall in the specificity of PRAME diagnostic utility. In addition, the number of studies involving difficult to classify melanocytic neoplasms and PRAME staining is small. Studies to determine whether PRAME expression in malignant and non-malignant melanocytic lesions such as dysplastic nevi, melanoma, and Spitz nevi correlates with the results of genomic testing are also small in number with some conflicting results. Lezcano et al. recently found 90% concordance between PRAME immunohistochemistry and cytogenetic study results in diagnostically difficult melanocytic neoplasms (including atypical Spitz tumor/nevus versus spitzoid melanoma, dysplastic nevus versus melanoma, and traumatized or mitotically active nevus versus melanoma) and concluded that it may be a useful ancillary test in this subset (5). Contrastingly, Raghavan et al. concluded that caution must be exercised when interpreting the results of PRAME immunohistochemistry in spitzoid neoplasms (6). Googe et al. found that while the majority of invasive melanomas in their study were PRAME positive (either focally or diffusely), 16% were entirely PRAME negative, raising further concerns over the reliability of PRAME (7). They also found 73% of Spitz nevi in their sample to be PRAME negative.

An additional area of discrepancy in the literature is the interpretation of PRAME positivity, with multiple approaches documented. The predominant approach seems to be that of Lezcano et al. which utilizes a scale of 0 to 4+ to grade the percentage of PRAME positive melanocytes, with 4+ representing “diffusely positive” with greater than 75% of melanocytes staining for PRAME (8). Googe et al. additionally commented on categorization as focally or diffusely positive PRAME staining, with 1+ to 3+ staining considered focally positive and 4+ considered diffusely positive (7). Umano et al. broke from the precedent of the 0–4+ PRAME positivity scale, and instead utilized a scale of 1+ to 3+, with 1+ considered slightly positive to 3+ considered intense positivity; they also commented on the location of the PRAME positive cells as junctional versus intradermal (9). Meanwhile, Raghavan et al. defined greater than 60% of positively staining melanocytes as PRAME positivity and also commented on intensity of the stain (6).

The purpose of this study is to contribute to the existing scientific literature, which at present demonstrates some caution over the utility and reliability of the use of PRAME as a screening or ancillary test for melanocytic lesions, particularly with regard to difficult spitzoid melanocytic neoplasms.

## 2. Methods

Samples were collected from the slide archive at Pinkus Dermatopathology Laboratory under an IRB-approved protocol based on a previous diagnosis of atypical spitz nevus, spitz nevus, spitzoid melanoma, or atypical compound melanocytic neoplasm. Exclusion criteria included insufficient tissue sample for study or absence of prior FISH cytogenetic testing. Inclusion criteria were biopsy specimens with a previous diagnosis of spitzoid melanoma, atypical spitzoid neoplasms or Spitz nevus with previous FISH testing. Archived cases which had undergone FISH testing were reviewed. Classical Spitz nevi in children which typically do not require ancillary testing were not included. Cases with a spitzoid morphology including epithelioid melanocytes, epidermal hyperplasia and clefting of the epidermis around the junctional nests but lacking sharp lateral circumscription or maturation with depth which had been sent for ancillary FISH testing were included. All lesions had a dermal component that showed a lack of maturation with depth. Spindle cell nevus of Reed and desmoplastic spitz nevi were not included. The final diagnosis was made by a combination of histologic findings and FISH results. These included Spitz nevi in adults, atypical Spitz nevi/atypical spitz tumors, and spitzoid melanomas.

A total of 83 spitzoid and atypical compound neoplasms were included for study. All had previously had FISH cytogenetic testing for melanoma performed. PRAME immunohistochemistry was performed on all samples, with nodular melanoma used as a control. Four micrometer tissue sections were treated with high pH 8 epitope retrieval for 10 min. The sections were stained with PRAME (Cell Marque clone EP46, Rocklin CA) for 15 minutes and detected using the Leica Bond III system with red chromogen (Deer Park, Ill). p16 staining was also performed in 21 of the cases.

### 2.1. PRAME immunohistochemistry

The staining pattern for PRAME antibody was investigated in non-malignant and difficult to diagnose melanocytic lesions, predominately those with spitzoid features including nests of epithelioid or spindle cell melanocytes, clefting around nests of melanocytes and epidermal hyperplasia. We correlated and compared PRAME results with previously obtained FISH analyses, as well as with staining in nodular melanoma and normal skin as positive and negative controls, respectively.

Investigators were blinded to the corresponding FISH results of each sample when quantifying the percentage of melanocytes staining positively for PRAME. The number of PRAME positive staining melanocytes in a square millimeter were counted in each sample by two independent researchers and then classified into a five-part scale based on the precedent set by Lezcano et al. (8). Samples with zero positively staining melanocytes were classified as negative (0); samples with staining of greater than zero through 25% of tumor cells are classified as 1+, staining of greater than 25% through 50% of tumor cells is considered 2+, staining of greater than 50% through 75% of tumor cells staining is 3+, and greater than 75% or more melanocytes staining is labeled as 4+ or “diffuse.”

**Figure 1:**
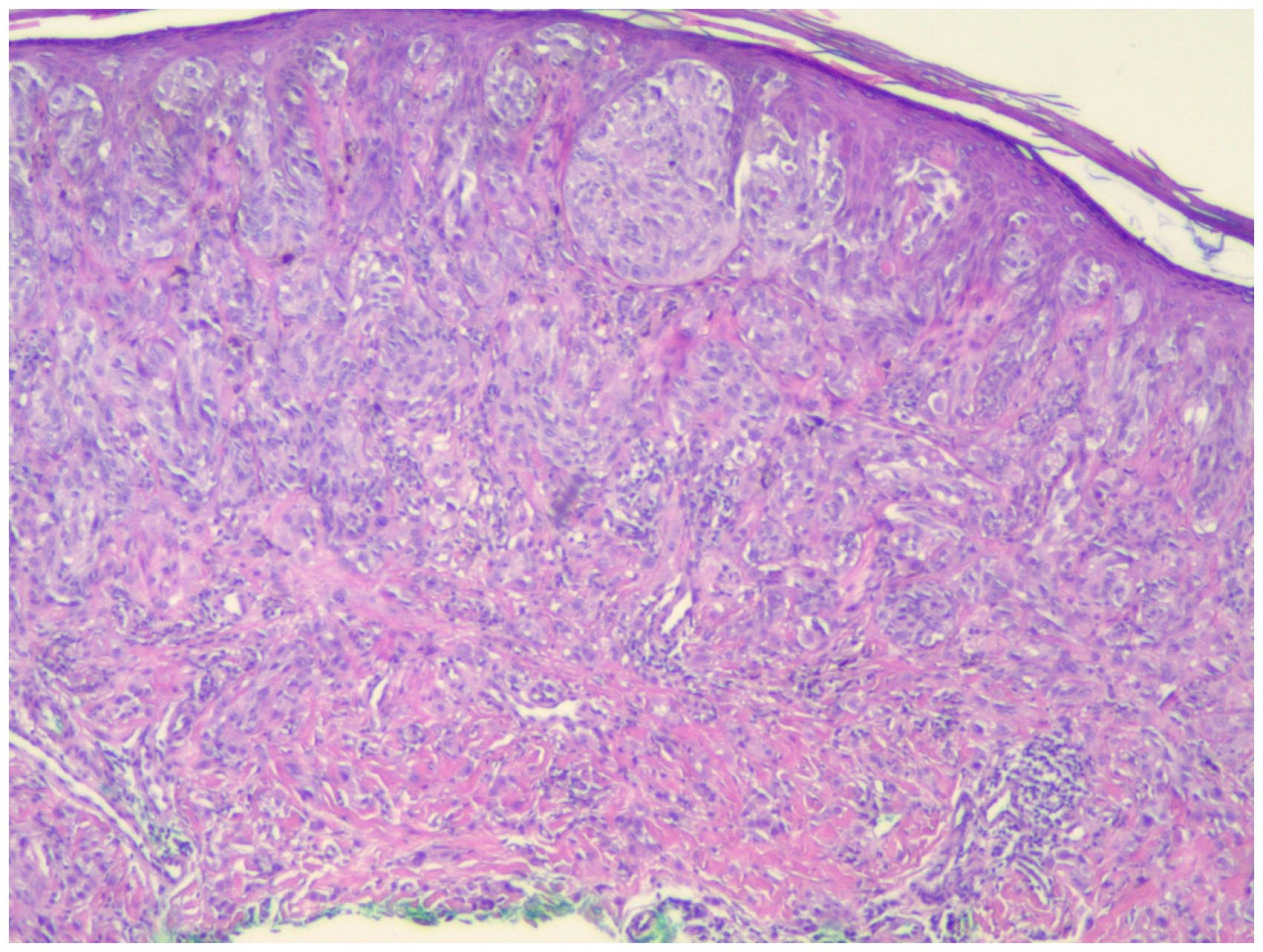
This spitzoid melanoma failed to stain with PRAME but was positive by FISH (H&E; 40×).

**Figure 2:**
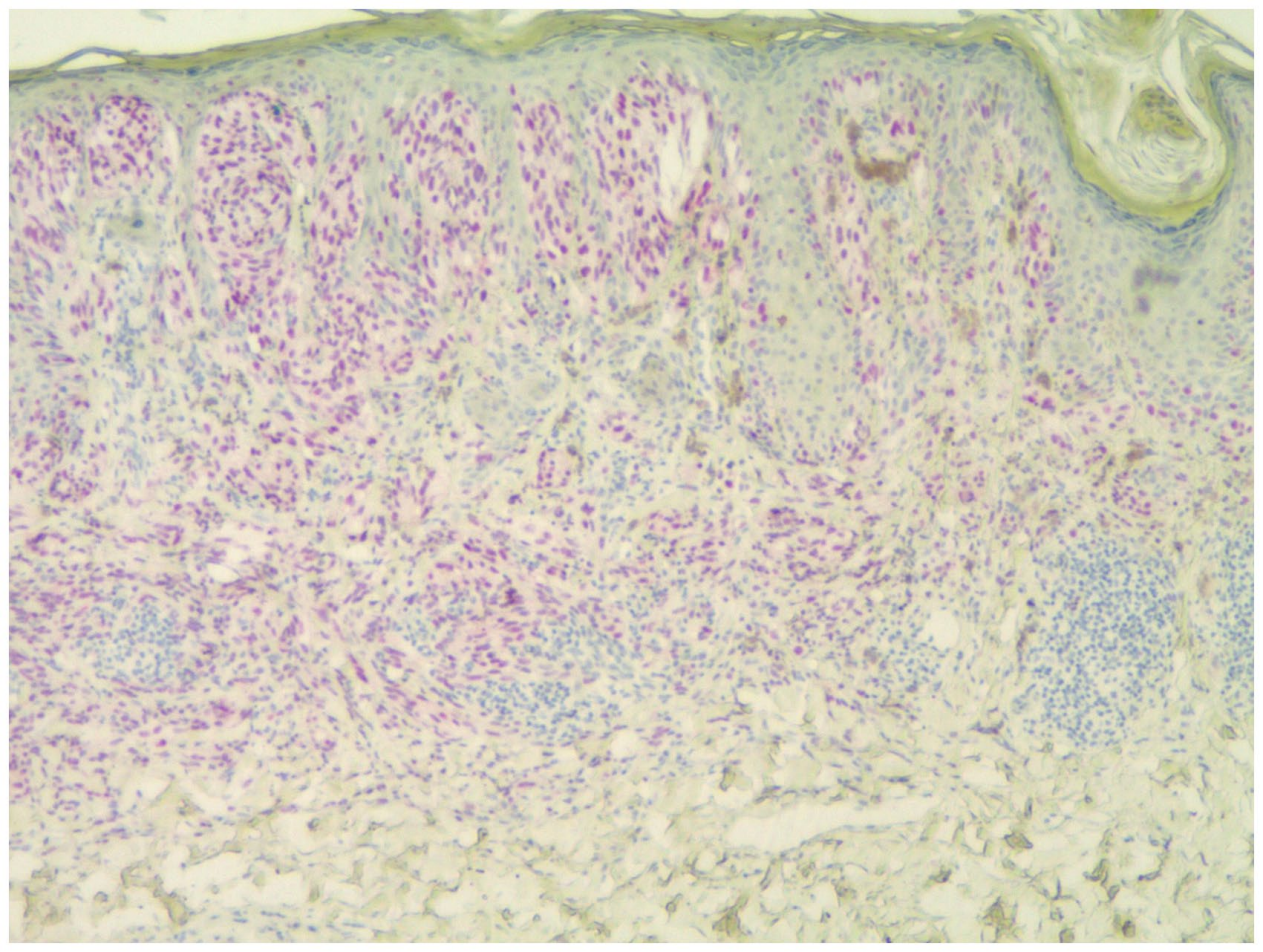
Spitzoid melanoma showing 3+ staining by PRAME (PRAME, 40×).

**Figure 3:**
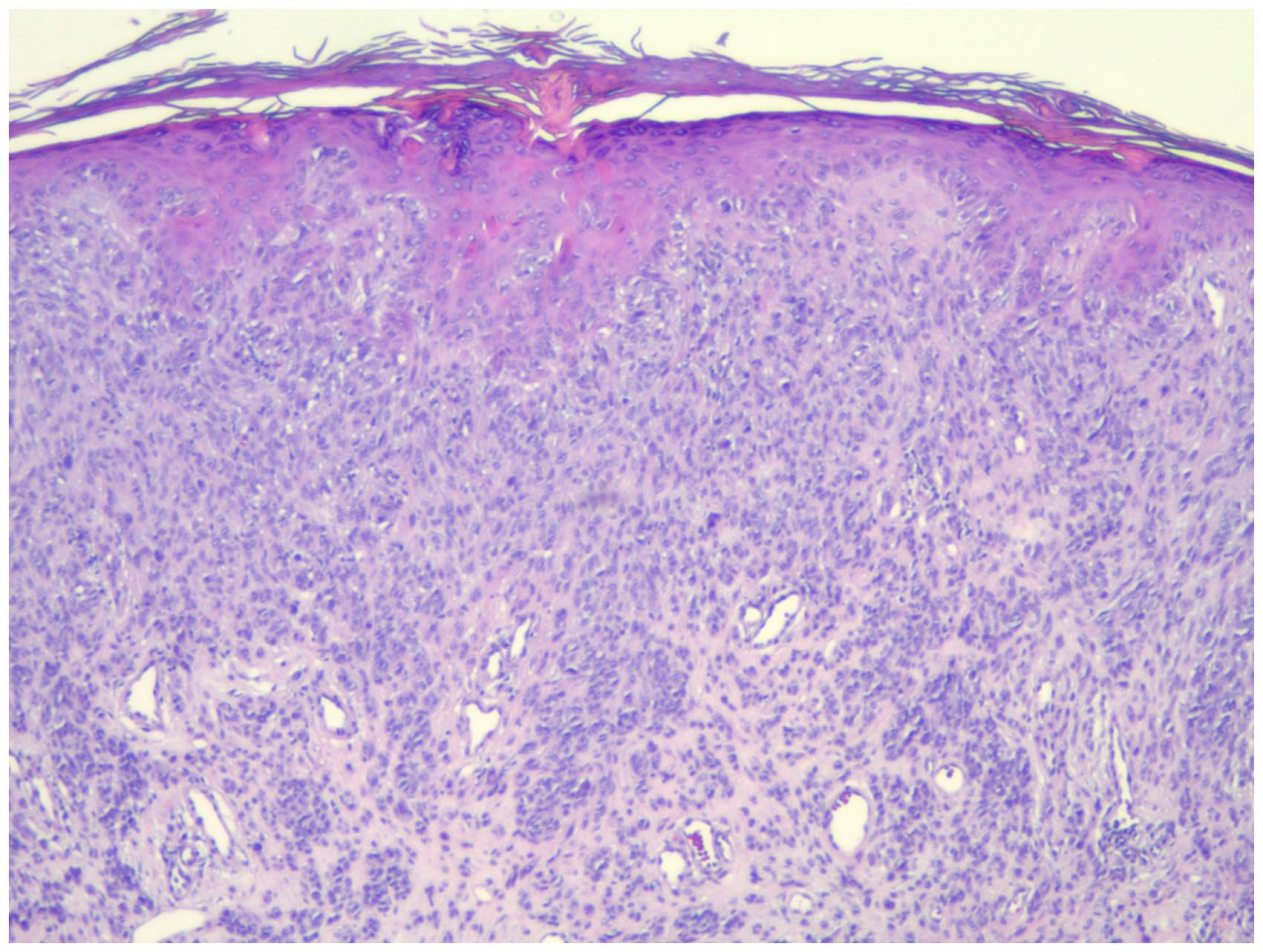
This spitz nevus stained positive for PRAME but was negative for FISH (40×).

**Figure 4:**
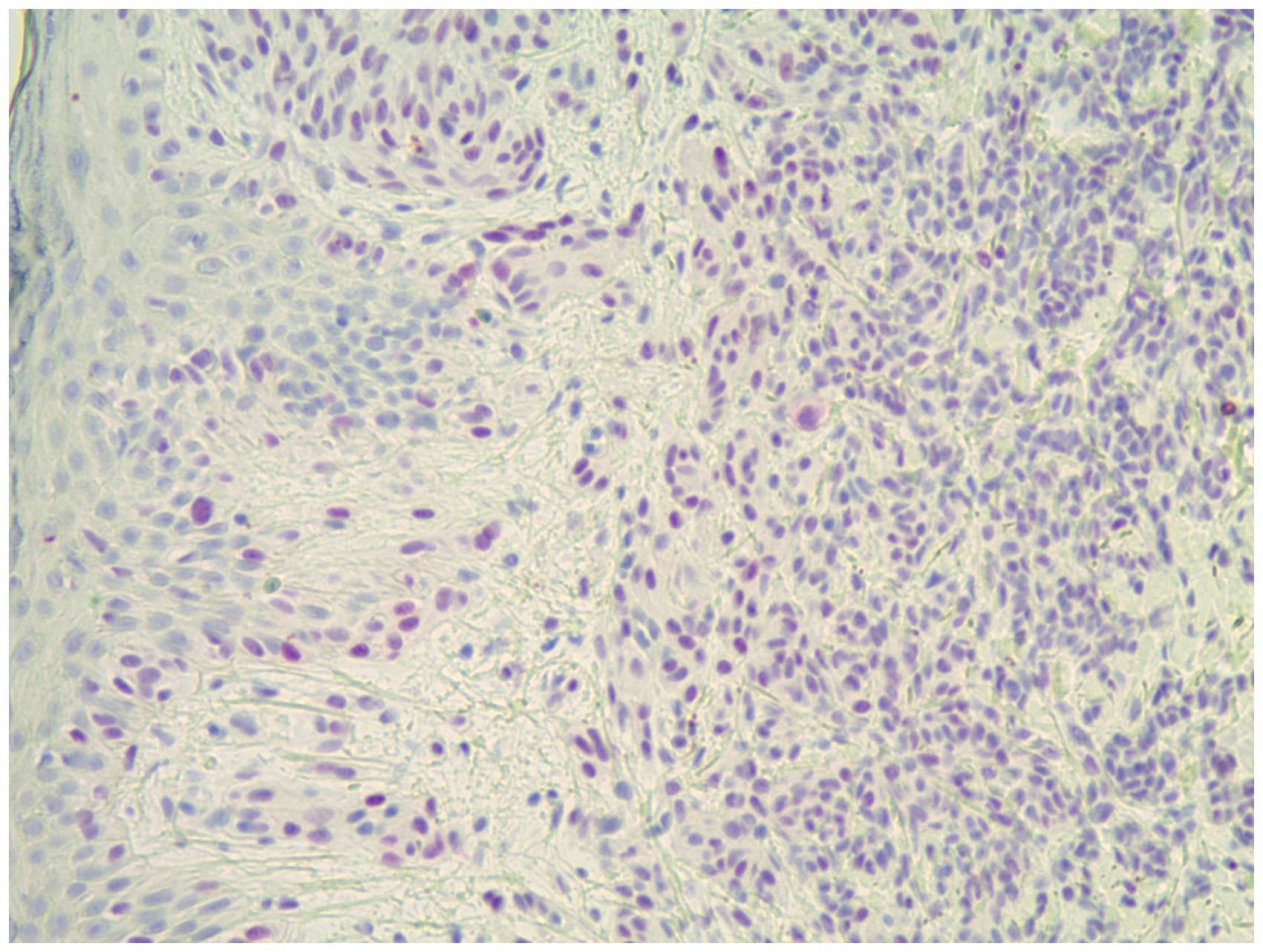
Strong positive PRAME staining in a spitz nevus which was negative for all FISH markers (40×).

**Figure 5:**
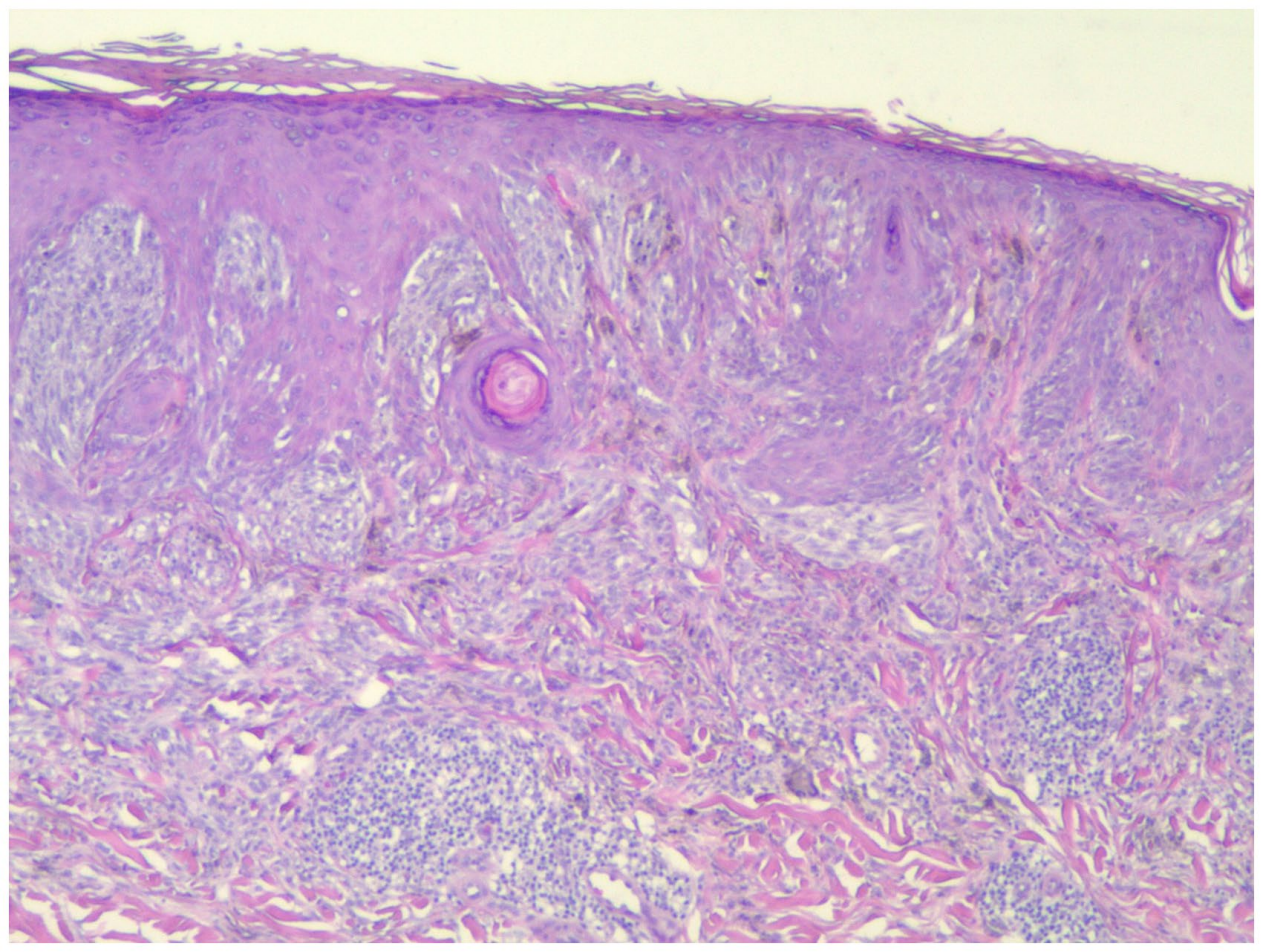
This spitzoid melanoma showed positive PRAME staining and was positive by FISH (H&E; 40×).

Individuals whose PRAME results were 0 or 1+ were classified as PRAME negative, as in Figure 1; those whose results were 2+–4+ were classified as PRAME positive, as in Figures 2–5.

### 2.2. Fluorescence *in situ* hybridization

The PRAME scale results were then correlated with the genomic testing results for each sample. Each sample had a pre-existing NeoSITE^™^ Melanoma FISH analysis performed by NeoGenomics, which included the genes RREB1 (6p25), cMYC (8q24), CDKN2A (p16)/CEN9, and CCND1 (11q13). The high stringency cutoff for a positive result with this test is >29% for any probe, whereas the low stringency cutoffs for “borderline positive” results are less than 29% of cells with RREB (6p25) gain but greater than 16%, cMYC (8q24) gain in greater than 10%, CDKN2A (p16)/CEN9 homozygous deletion in greater than 10%, and CCND1 (11q13) gain in greater than 19% of cells. Negative results are defined as RREB (6p25) <16%, cMYC (8q24) <10%, CDKN2A (p16)/CEN9 < 10%, and CCND1 (11q13) <19%. Gerami et al. have found the high stringency cutoffs to be 94% sensitive and 98% specific in differentiating between benign and malignant melanocytic neoplasms, but only 70% sensitive in spitzoid neoplasms (10).

Data analysis was conducted in the R statistical programming language (version 4.1.2).

## 3. Results

Eighty-three spitzoid and atypical compound neoplasms were ultimately included in this study. Twenty-seven were ultimately categorized as malignant by FISH. Patient ages ranged from 2–79 with a mean age of 33.2 and a median age of 30.0.

The body-site distribution of the samples was as follows: 21.7% from the head and face, 22.9% from the trunk, 30.1% from the upper extremity (including shoulder), and 25.3% from the lower extremity.

p16 staining was performed in 21 cases, with loss of p16 observed in two cases which were diagnosed as melanoma. These two cases showed 0 and 1+ PRAME staining but received FISH staining over the high stringency cut off values for malignancy. In the cases in which p16 expression was retained there were 7 melanomas and 12 Spitz or atypical Spitz nevi.

FISH for melanoma had been previously performed in every case, and PRAME IHC was performed on all cases.

Of the 83 samples, 56 were FISH negative, and 27 were FISH positive. The distribution with respect to PRAME is summarized in Tables 1–3. Out of the 83 specimens graded on the PRAME 0–4+ scale, 49 were PRAME 0, 13 stained 1+, five stained 2+, three stained 3+, and 13 stained 4+, as demonstrated in Table 1. If a lower cutoff is utilized for PRAME positivity, defining positivity as staining 2+, 3+, or 4+, 21 out of the 83 samples would be PRAME positive, and 62 would be PRAME negative (defined as PRAME 0 or 1+), as demonstrated in Table 2.

**Table 1:** Distribution of PRAME staining on the five-point scale established by Lezcano et al. with corresponding FISH results (FISH benign corresponds to negative FISH, FISH malignant corresponds to positive FISH).

PRAME staining results	FISH benign	FISH malignant	Total
0	38	11	49
1+	5	8	13
2+	4	1	5
3+	3	0	3
4+	6	7	13
Total	56	27	83

**Table 2:** Distribution of PRAME staining when categorized as negative (PRAME 0 or 1+) or positive (PRAME 2+, 3+, or 4+) with corresponding FISH results (FISH benign corresponds to negative FISH, FISH malignant corresponds to positive FISH).

PRAME staining	FISH benign	FISH malignant	Total
Negative	43	19	62
Positive	13	8	21
Total	56	27	83

**Table 3:** Sensitivity, specificity, positive predictive value (PPV), and negative predictive value (NPV) of PRAME in our sample, positing PRAME as the experimental test in comparison to FISH.

	*n*/*N*	Pct	(95% CI)
Sensitivity	8/27	29.6%	(13.8, 50.2%)
Specificity	43/56	76.8%	(63.6%, 87.0%)
PPV	8/21	38.1%	(18.1, 61.6%)
NPV	43/62	69.4%	(56.3, 80.4%)

Key: *n*/*N*, numeric; Pct, percent.

In this dataset, the sensitivity of PRAME for malignant lesions is 29.6%, with a 95% confidence limit of 13.8 to 50.2%, as demonstrated in Table 3. The estimated sensitivity is too low to indicate a useful marker. In addition, the confidence limit is very wide and includes 50%, indicating an imprecise estimate.

The specificity of PRAME for malignant lesions is 76.8% with a confidence limit of 63.6–87.0%. Estimated specificity is near the level that indicates usefulness, but the confidence limit is wide, again indicating lack of precision.

In our dataset, a subgroup analysis was performed looking at the differences in specificity, sensitivity, positive predictive value (PPV), and negative predictive value (NPV) when the threshold for positivity was adjuste, as in Tables 4, 5. When adjusted for a positivity threshold of 3+ to 4+ PRAME staining, our sensitivity lowered slightly from 29.6 to 25.9% (from 8/27 to 7/27), however the specificity increased from 83.9% (47/56) to 89.3% (50/56), respectively, as shown in Table 5. Similarly, the PPVs and NPVs increased as seen in the tables below, suggesting that by raising the threshold of positivity we were able to eliminate false negatives without sacrificing the capture of true positives.

**Table 4:** Subgroup analyses adjusting for different positivity thresholds: positivity defined as anything staining greater than PRAME 1+, PRAME 2+, or 3+ accordingly.

FISH result	>PRAME 1+	>PRAME 2+	>PRAME 3+
FISH Benign	43 Neg/13 Pos	47 Neg/9 Pos	50 Neg/6 Pos
FISH Malignant	19 Neg/8 Pos	20 Neg/7 Pos	20 Neg/7 Pos

**Table 5:** Subgroup analyses adjusting for different positivity thresholds: positivity defined as anything staining greater than PRAME 1+, PRAME 2+, or 3+ accordingly with adjusted sensitivity, specificity, positive predictive value (PPV), and negative predictive value (NPV) of PRAME in our sample at these different cutoffs, positing PRAME as the experimental test in comparison to FISH.

	>PRAME 1+	>PRAME 2+	>PRAME 3+
Statistic	n/N Pct	n/N Pct	n/N Pct
Sensitivity	8/27 29.6%	7/27 25.9%	7/27 25.9%
Specificity	43/56 76.8%	47/56 83.9%	50/56 89.3%
PPV	8/21 38.1%	7/16 43.8%	7/13 53.8%
NPV	43/62 69.4%	47/67 70.1%	50/70 71.4%

Key: *n*/*N*, numeric; Pct, percent.

## 4. Discussion

Immunohistochemical stains including HMB-45, Mart-1, Ki-67, and p16 are commonly utilized in the diagnosis of difficult melanocytic lesions. PRAME (PReferentially expressed antigen in melanoma) is a cancer testis antigen found to be overexpressed in melanoma. In large studies approximately 90% of primary melanomas showed nuclear PRAME staining while 98% of nevi are negative (11). O’Connor et al. reviewed PRAME staining in 101 benign melanocytic nevi and 42 malignant melanomas (12). They showed that using a 75% of cells staining score was associated with a sensitivity of 0.63, a specificity of 0.97 and an accuracy rate of 87% (12). However, due to the relative rarity of spitzoid melanoma, there is less data available for these neoplasms. Smaller studies using PRAME have been reported. Chen et al. reported 5 cases of spitzoid melanomas of which 3 (60%) stained diffusely positive for PRAME. 11 Koh et al. also utilized PRAME staining in spitzoid neoplasms (13). In their study of 35 lesions, 20% of Spitz nevi showed staining of greater than 75% of cells while 82% of spitzoid melanomas were similarly positive (13). However neither of these studies correlated FISH results with PRAME staining. Fluorescence *in-situ* hybridization (FISH) utilizing probes for genes RREB1 (6p25), cMYC (8q24), CDKN2A (p16)/CEN9, and CCND1 (11q13) has been shown to be sensitive and specific for differentiating Spitz nevi from spitzoid appearing melanoma. Our study looked predominately at spitzoid melanocytic neoplasms in young adults which are a common diagnostic dilemma.

The risk of false negatives must be taken into account when determining the threshold for “diffusely positive.” As missing the diagnosis of melanoma is a very grave risk, we would recommend erring on the side of caution and utilizing a lower threshold for the percentage of PRAME positive cells considered as a positive test. However, in our study set, even by lowering our positivity threshold to PRAME 2+, we recaptured only 1 of the 20 samples that were identified as false negatives by FISH analysis; 8 of these FISH positive samples were quantified as PRAME 1+ and 11 did not stain for PRAME whatsoever, as in Figure 1. This poses a serious risk of utilizing PRAME alone in the diagnosis of melanoma in atypical spitzoid melanocytic neoplasms.

A screening test should have a higher sensitivity than that which we found in our study; had PRAME been used as a screening test to determine which cases warranted the more costly and time-consuming genetic testing in our study, only 8 out of the 27 or 29.6% of cases ultimately diagnosed as malignant after genetic testing would have been identified.

Despite the relatively small sample size of this study, there were 20 cases which would be classified as negative for PRAME on the 0–4+ scale but which were FISH positive, and ultimately were diagnosed as malignant, raising concern for the safety of utilizing PRAME immunohistochemistry as a screening test.

## 5. Conclusion

The differential diagnosis of spitzoid melanocytic neoplasms can be difficult. Ancillary testing including immunostaining for p16 and PRAME as well as fluorescence *in situ* hybridization have been utilized as diagnostic aids. In our study we sought to evaluate whether PRAME staining correlated with FISH results. We have concluded that PRAME immunohistochemistry does not show good correlation with FISH results in spitzoid melanocytic neoplasms, and ultimately, our study did not confirm its relevance as a screening tool. We also suggest, in line with Raghavan et al. that a lower threshold percentage of PRAME positive staining melanocytes might be utilized to increase the sensitivity of PRAME as a potential screening test. The lack of consensus in the literature on the appropriate percentage of positively staining melanocytes required for a lesion to be considered “diffusely positive” can also make it difficult to interpret the significance of this test between studies, although the majority follow the precedent set by Lezcano et al. However, the smaller sample size is a limitation of this study.

Although Lezcano et al. concluded that PRAME immunohistochemistry may still have use as an ancillary test as it is largely positive in melanomas and negative in benign lesions, our study showed some lack of concordance with FISH testing. We recommend that PRAME staining be interpreted in combination with other immunohistochemical results. Further study of the use of PRAME immunohistochemistry in these difficult to diagnose melanocytic neoplasms, particularly spitzoid neoplasms, is still warranted, particularly in the setting of larger datasets.

## Data availability statement

The raw data supporting the conclusions of this article will be made available by the authors, without undue reservation.

## Ethics statement

The studies involving humans were approved by Wayne State University Institutional Review Board. The studies were conducted in accordance with the local legislation and institutional requirements. The human samples used in this study were acquired from a by-product of routine care or industry. Written informed consent for participation was not required from the participants or the participants’ legal guardians/next of kin in accordance with the national legislation and institutional requirements.

## Author contributions

EW: Data curation, Investigation, Project administration, Writing – original draft, Writing – review & editing. DM: Investigation, Writing – original draft, Writing – review & editing, Conceptualization, Data curation, Methodology, Project administration, Supervision. SU: Investigation, Writing – original draft, Writing – review & editing, Funding acquisition. RS: Data curation, Formal analysis, Methodology, Software, Writing – review & editing. JA: Data curation, Formal analysis, Methodology, Software, Writing – review & editing.
